# Desired improvements of working conditions among medical assistants in Germany: a cross-sectional study

**DOI:** 10.1186/s12995-019-0237-x

**Published:** 2019-06-01

**Authors:** Jessica Scharf, Patricia Vu-Eickmann, Jian Li, Andreas Müller, Stefan Wilm, Peter Angerer, Adrian Loerbroks

**Affiliations:** 1 Institute of Occupational, Social and Environmental Medicine, Centre for Health and Society, Faculty of Medicine, University of Düsseldorf, Universitätsstr. 1, 40225 Düsseldorf, Germany; 2 Department of Environmental Health Sciences, Fielding School of Public Health, School of Nursing, University of California Los Angeles, Los Angeles, USA; 3 Institute of Psychology, Work and Organizational Psychology, University of Duisburg-Essen, Universitätsstr. 2, 45, ,141 Essen, Germany; 4 Institute of General Practice, Centre for Health and Society, Faculty of Medicine, University of Düsseldorf, Werdener Str. 4, 40227 Düsseldorf, Germany

**Keywords:** Health occupation, Medical assistants, Working conditions, Needs assessment, Cross-sectional study

## Abstract

**Background:**

In outpatient care in Germany, medical assistants (MAs) are the contact persons for patients’ concerns and their working conditions are relevant to their own health and the provided quality of care. MAs working conditions have been described as precarious leading to high levels of work stress. Consequently, we aimed to examine MAs’ needs for work-related improvements.

**Methods:**

We surveyed 887 employed MAs between September 2016 and April 2017. A 20-item questionnaire measured desired improvements. To measure correlations between variables we computed a matrix of tetrachoric correlations for binary variables and performed an exploratory factor analysis. We ran ordinal logistic regression models employing 11 independent variables to examine determinants of needs.

**Results:**

A total of 97.3% of the participants expressed any need and, on average, 10.27 needs were reported. Most frequently, needs were expressed related to a higher salary (87.0%), less documentation (76.0%) and more recognition from society (75.4%). Exploratory factor analysis suggested three dimensions of needs for work-related improvements (i.e. working conditions, reward from the supervisor and task-related independence). Ordinal logistic regression models only identified determinants for the outcome variable task-related independence, which was more frequent in those with longer work experience or in a leadership position.

**Conclusions:**

The high prevalence of desired workplace-related improvements among MAs highlights the relevance of modifying their working conditions. The fact that we found only few determinants signals that there are no specific high-risk subgroups, but interventions to improve MAs’ working conditions should target the entire MA population.

**Electronic supplementary material:**

The online version of this article (10.1186/s12995-019-0237-x) contains supplementary material, which is available to authorized users.

## Introduction

Medical assistants (MAs; in German: “Medizinische Fachangestellte”) represent the largest occupational group in outpatient care in the German health care system. Medical assistants usually provide basic clinical and administrative assistance in outpatient physician practices [1]. A large proportion of tasks (e.g., related to standardized diagnostic procedures, such as taking blood samples, ECG or spirometry) are assigned to MAs and supervised by physicians. Further, MAs act as the first contact persons for patients and their queries or complaints and make significant contributions to patient satisfaction, patient safety and the quality of the doctor-patient communication [2–4]. These quality of care indicators are, amongst others, determined by the psychosocial working conditions of health care staff [5]. MAs’ working conditions have been characterized as precarious, which includes low wages, irregular working hours [6], high workloads and the requirement to handle unforeseeable incidents at the workplace [7]. As a corollary, MAs experience high levels of work stress [8, 9]. While interpersonal difficulties with supervisors (i.e. mostly physicians) and colleagues are often perceived as an additional burden, social support from colleagues is also perceived as a work-related resource alleviating work stress [7]. Overall, MAs’ working conditions have received little research attention to date. This is contrasted by abundant research on the working conditions of some other health care professions, in particular physicians and nurses [10, 11]. As the working contexts and tasks of nurses and MAs differ considerably, it cannot be assumed that insights into the work stress among nurses can be readily generalized to MAs. As mentioned above, MAs are involved in the long-term management of patients and likely have more frequent and closer interpersonal contact with patients, who may in some instances act reluctantly, very demanding and impolitely [12]. Furthermore, in contrast to nurses, MAs’ duties, such as having a large number of very short interactions with patients over the telephone each day, have been found to be associated with exceptionally high psychological demands and feelings of low job control [13]. Furthermore, compared to nurses, MAs’ tasks may range from administrative tasks to the execution of various medical procedures as delegated by the physician. As a consequence the match between the MAs preferred tasks and the actual tasks may be low. Such mismatches have been found to be associated with reduced job satisfaction and poor mental health [14]. Furthermore, in contrast to hospital settings, outpatient practices mainly have small teams and their physician acts as both the formal supervisor and employer of MAs. As a consequence, the working climate, career prospects and career development of MAs may strongly depend on the characteristics and leadership style of that physician. Additionally, if a team member is absent from work due to sickness, management of the additional workload by the absent employee is obviously more challenging for smaller teams, such as in outpatient practices.

Therefore, research into the specific working conditions of MAs is crucial due to the close link of adverse working conditions with poor health outcomes (e.g. poor self-rated health and symptoms of depression or anxiety), increased self-reports of major medical errors and intentions to leave the profession, as documented by a recent study from our group [9].

A proper needs assessment represents the starting point for the development, implementation and evaluation of interventions according to well-established health promotion frameworks (e.g. PRECEDE-PROCEED) [15]. Having identified several types of key occupational needs and desired improvements in our previous qualitative study [7] we now sought to quantify those needs in terms of their prevalence and potential determinants in the present study. Needs may be conceptualized in terms of the so called person-environment fit theory, which describes the (mis)match between characteristics of individuals and their work environments, in particular the match between the needs of a person and the supplies in the environment pertaining to the person’s needs contribute to job satisfaction [16]. In the literature, different definitions of a need can be found. While there are definitions of needs from a pragmatic (i.e., *whatever is required for health or comfort*) or economical viewpoint (i.e., *the ability of people to benefit from health care provision*), we adopt a societal viewpoint of a need (i.e., *felt, expressed, normative and comparative needs*) [17]. Especially, we focus on felt needs (equal with subjective “wants”) as well as expressed needs (where “felt needs” become a demand) to determine the gap between the current condition and a wanted condition from a subjective viewpoint [18, 19].

Based on the presented theoretical background we aim to address the following research questions:

1. What is the prevalence of work-related intervention needs among medical assistants?
2. How can those work-related intervention needs be clustered?
3. Are the clustered work-related intervention needs associated with sociodemographic or practice-related determinants?

## Materials and methods

### Sample

Data collection was carried out between September 2016 and April 2017. Participants could choose between a postal and an online survey to provide their data. MAs were recruited throughout Germany with the support of multipliers and various communication channels, as detailed elsewhere [9]. Briefly, the study was advertised in the members’ magazine of the Association of Medical Professionals (VMF e.V., which represents MAs) as well as published on the homepages of the Association of Statutory Health Insurance Physicians and State Medical Association. Furthermore, physician offices and professional MA schools were contacted. Eligible participants were either on current training or held a degree as a MA. The study was approved by the Ethics Committee of the Medical Faculty of the Heinrich-Heine-University of Düsseldorf (ethic registration number: 4778).

### Questionnaire

The questionnaire covered socio-demographic variables, data on occupational activity and current employment, job satisfaction, health status, involvement in patient care and desired work-related improvements. The questionnaire section on desired work-related improvements has been developed by our group based on insights from prior qualitative interviews with 26 MAs [7]. The final questionnaire assessing work-related intervention needs comprised 20 items (see Table 2). The topics that were covered included working conditions, work organization, rewards from the supervisor, task-related independence, working climate and leadership. Items were presented as statements and the following response categories were provided: “Y*es, I would like this”*, “*This need has already been met”* and “*No, I don’t need this”.* This response format had been developed and used in prior research [20]. Basic psychometric properties of this instrument will be presented in this report (see below).

### Statistical analyses

First, we carried out descriptive analyses by determining the prevalence of each work-related improvement need based on the above-mentioned three response categories. Second, to identify dimensions of needs, an exploratory factor analysis (EFA) was performed (i.e. varimax rotation). Therefore, to specifically capture expressed needs, we dichotomized the response categories into “Y*es, I would like this”* (scored as 1) versus “*This need has already been met”* or “*No, I don’t need this”* (scored as 0). To measure correlations between variables (i.e., the correlation matrix) we computed a matrix of tetrachoric correlations for binary variables, as we used only two response categories in our analysis and it has been shown that fewer than four response categories lead to decreasing reliability and validity when applying an EFA [21–23]. The aim of the EFA was to reduce the number of variables by combining variables into independent factors. Those factors were considered to represent subscales, which we will refer to as *dimensions* in the remainder of the paper. We calculated Cronbach’s α coefficients to assess the internal consistency of each dimension.

Third, we sought to identify determinants of needs. The selection of potential determinants (i.e. the independent variables) was based on prior studies on working conditions in primary care practice teams [8, 24, 25]. Data on determinants was entirely based on reports of the participating MAs and included:

- Age in years, which was categorized into tertiles (i.e. 18–35 years, 36–45 years, ≥ 46 years)
- Marital status which differentiated between being single or in a partnership
- Three gross income categories (≤1499€, 1500-1999€, ≥2000€)
- Total years employed in the MA profession (including years in training), categorized based on tertiles (i.e. ≤10 years, 11–20 years, ≥21 years)
- Physician office type, dichotomized into working for a general practitioner or any type of specialist
- Employment status was either full-time or part-time/mini-job
- Self-reported leadership responsibility was categorized as “yes” or “no”
- The number of medical assistants in the employing practice was categorized based on tertiles (1–3 MAs, 4–6 MAs, ≥ 7 MAs)
- The number of physicians in the practice was categorized based on tertiles (1 physician, 2 physicians, ≥ 3 physicians)
- Practice size was calculated by the number of MAs and physicians in a practice (1–5 employees, 6–10 employees, ≥ 11 employees)
- Practice location was defined as rural area (< 20,000 inhabitants), small city (20,000 to 100,000 inhabitants) or large city (> 100,000 inhabitants)

The dependent variables were dimensions of desired work-related improvements. To create such variables, we calculated dimension-specific sum scores (i.e. the respective number of desired work-related improvement), which were categorized into three groups:

- No desired work-related improvement (a score 0 on the respective subscale)
- Some desired work-related improvements (a score higher than 0, but lower than the maximum score)
- Many desired work-related improvements (maximum score on the respective needs subscale).

This three-level categorization was chosen, first, because the dichotomization of needs had implied information loss (e.g. maximum needs vs lower; or any versus none); and second, because the categorization we used to some extent harmonized the outcomes which had varying score ranges (i.e. the lowest category reflecting the lowest score and the highest category the maximum score).

Associations between potential determinants (i.e. independent variables) and dimensions of desired work-related improvements (i.e. dependent variables) were examined by ordinal logistic regression. The results of the calculated ordinal logistic regressions are shown as odds ratio (OR) with 95% confidence intervals (CIs).

For each potential determinant and each outcome variable unadjusted as well as age-adjusted models were initially computed. We did not further adjust for gender since our study population was almost exclusively comprised women (see below). Next, ordinal logistic regressions with backward selection were run to identify statistically independent determinants. Starting with all variables in the model, all variables above the model fit criterion of 0.2 – which is a recommended threshold [26] - were excluded from the model. This process was repeated until no further variables could be excluded. All analyses were carried out using SAS.

## Results

The results section is organized in line with the three research questions presented in the introduction. After a presentation of the characteristics of the study sample, we will first show the distribution of the work-related intervention needs. Second, results from factor analysis are presented to identify if/how the work-related intervention needs clustered. Finally, ordinal logistic regressions (with backward selection) estimates are shown which analyze if the clustered work-related intervention needs are associated with sociodemographic or practice-related determinants.

### Study sample

A total of 994 MAs participated. Due to the research focus of this report, the sample was restricted to those in employment (*n* = 887). Virtually all participants were female (98.4%) with a mean age of 39.3 years (standard deviation (SD) = 11.4, see Table 1). About every second participant was in a partnership (50.7%). Most MAs held a secondary school certificate (74.6%) and worked fulltime (59.2%) with a monthly gross salary of up to 1999€ (58.5%). As much as 63.4% of the MAs had less than 20 years of work experience. The proportion reporting to work for a general practitioner or a specialist was almost equal (47.7 and 52.3%, respectively). The mean reported practice size in terms of staff members was 9.0 (SD = 5.4). Most practices were located in small cities (42.0%) followed by large cities (38.9%) and rural areas (19.1%).

**Table 1:** Description of the sample (*n* = 887)

Characteristics	
Age, mean (standard deviation)	39.3 (11.4)
18–35, n (%)	264 (30.1)
36–45, n (%)	312 (35.5)
≥ 46, n (%)	302 (34.0)
Female, n (%)	865 (98.4)
Marital status, n (%)	
In a partnership	445 (50.7)
Single	433 (49.3)
Highest school degree, n (%)	
Low^a^	52 (5.9)
Intermediate^b^	653 (74.6)
High^c^	170 (19.4)
Gross salary (€), n (%)	
≤ 1499	291 (33.6)
1500€-1999	216 (24.9)
≥ 2000	360 (41.5)
Years in job, mean (SD)	17.3 (11.3)
Years in job, n (%)	
0–10	293 (35.1)
11–20	236 (28.3)
≥ 21	305 (36.6)
Practice type, n (%)	
General practitioner	345 (47.7)
Specialist	379 (52.3)
Employment status, n (%)	
Full-time	510 (59.2)
Part-time/Mini-job	351 (40.8)
Leadership position (yes), n (%)	421 (48.0)
Number of MAs in the employing practice, n (%)	
1–3	288 (34.6)
4–6	317 (38.1)
≥ 7	227 (27.3)
Number of practitioners in the employing practice, n (%)	
1	262 (31.5)
2	250 (30.0)
≥ 3	320 (38.5)
Practice size, mean (SD)	8.98 (5.36)
Practice location, n (%)	
Large city	343 (38.9)
Small city	370 (42.0)
Rural area	168 (19.1)

^a^Low: secondary modern school qualification (‘Haupt−/Volksschulabschluss’)

^b^intermediate: secondary school level I certificate (‘Mittlere Reife’)

^c^high: general qualification for university entrance (‘Abitur’) or entrance qualification limited to universities of applied sciences (‘Fachhochschulreife’)

### Distribution of the work-related intervention needs

Desired work-related improvements can be found in Table 2. The most frequently expressed need pertained to a higher salary (87.0%). At least three out of four MAs expressed a) the need for less documentation during work (76.0%), b) for more recognition from society (75.4%) as well as c) for educational opportunities related to organizational leadership for supervising physicians (75.1%). Two thirds reported needs pertaining to less multitasking at work. Overall, there were 11 needs with at least 50% agreement among MAs. Irrespective of the particular type of need, 97.3% of the participants expressed any need and, on average, 10.3 needs were reported (SD = 3.9). Occupational features which were reported not to be needed pertained to, for instance, making home visits (63.7%) and to advise patients about their disease (51.9%). Moreover, additional breaks (51.2%) and different opening hours of the practice/clinic (49.9%) were also often reported not to be needed by the MAs.

**Table 2:** Desired work-related improvements among medical assistants

Item	Yes, I would like this, n (%)	This need has already been met, n (%)	No, I don’t need this, n (%)
I would like to have more responsibility in my job.	230 (26.3)	448 (51.3)	196 (22.4)
I would like to have a greater scope of action and freedom of choice.	356 (40.9)	358 (41.1)	157 (18.0)
I would like to independently advise patients about their disease.	236 (27.1)	183 (21.0)	453 (51.9)
I would like to make home visits.	112 (12.9)	203 (23.4)	552 (63.7)
I would like to have more educational opportunities.	478 (54.9)	304 (34.9)	89 (10.2)
I would like to have additional breaks.	346 (39.9)	78 (9.0)	444 (51.2)
I would like to have different opening hours of the practice/clinic.	368 (42.3)	67 (7.7)	434 (49.9)
I would like to have more staff at my workplace.	488 (55.7)	89 (10.2)	299 (34.1)
I would like to work less hours.	466 (53.9)	43 (5.0)	356 (41.2)
I wish for more understanding by my supervisor.	528 (60.3)	207 (23.7)	140 (16.0)
I would like to have a better working climate.	400 (45.9)	274 (31.4)	198 (22.7)
I wish for improved interactions between colleagues.	330 (37.9)	297 (34.1)	243 (27.9)
I wish for more appreciation for my work from my supervisor.	531 (60.8)	236 (27.0)	106 (12.1)
I wish for more recognition for my work from the society.	654 (75.4)	69 (8.0)	144 (16.6)
I would like to have a higher salary.	759 (87.0)	85 (9.7)	28 (3.2)
I would like the physicians to have educational opportunities related to organizational leadership.	653 (75.1)	48 (5.5)	169 (19.4)
I would like to have less documentation in my day-to-day work.	659 (76.0)	19 (2.2)	189 (21.8)
I wish for a better organization of the practice/clinical procedures.	479 (54.7)	184 (21.0)	213 (24.3)
I would like to include the internet/ new media in my daily work.	277 (31.8)	306 (35.1)	288 (33.1)
I would like to have less multitasking.	585 (67.5)	26 (3.0)	256 (29.5)

Besides the above-mentioned needs, there were also needs that had already been met. Any need for more responsibility was reported to be already met by 51.3%, as was the need for a greater scope of action and freedom of choice (41.1%).

### Clustering of work-related intervention needs

After descriptive analyses, we performed the exploratory factor analysis (EFA). As mentioned above, the desired work-related improvement variables were dichotomized into *yes* (“Yes, I would like this”) versus *no* (“No, I don’t need this” or “This need has already been met”) for this purpose. Factors were identified based on the screeplot. Also factor loadings for the individual items had to exceed approximately 0.50 to be considered [27]. We initially ran an EFA with all 20 items. Ultimately, a total of 12 items remained after stepwise exclusion of items which failed to exceed the predefined factor loading threshold. The remaining 12 items clustered into 3 dimensions (see Table 3) labeled “working conditions” (5 items), “reward from the supervisor” (3 items) and “task-related independence” (4 items), respectively. The dimension “working conditions” (potential score range = 0–5; Cronbach’s α: 0.65) was composed of four desired work-related improvements regarding a lower workload, additional breaks, reduced multitasking, and the change of opening hours of the practice/clinic (see Table 3). “Reward from the supervisor” (potential score range = 0–3; Cronbach’s α: 0.70) captured the desire for more appreciation for one’s work from the supervisor, more understanding by one’s supervisor and a higher salary. The desire for a greater scope of action, more responsibility and advising patients about disease independently formed the dimension “task-related independence” (potential score range = 0–4; Cronbach’s α: 0.61).

**Table 3:** Results from exploratory factor analyses (i.e., factor loadings) ^a^

	Working conditions	Reward from the supervisor	Task-related independence
I would like to work less hours.	0.809		
I would like to have additional breaks.	0.698		
I would like to have less multitasking.	0.625		
I would like to have more staff at my workplace.	0.535		
I would like to have different opening hours of the practice/clinic.	0.471		
I wish for more appreciation for my work from my supervisor.		0.900	
I wish for more understanding by my supervisor.		0.861	
I would like to have a higher salary.		0.473	
I would like to have more responsibility in my job.			0.738
I would like to have a greater scope of action and freedom of choice.			0.715
I would like to independently advise patients about their disease.			0.660
I would like to make home visits.			0.523

^a^only items with factor loadings approximately 0.5 or higher are shown

### Association of sociodemographic and practice-related determinants with work-related intervention needs

As presented in Table A1-A3 (see Additional files 1, 2 and 3), unadjusted ordinal logistic regression models suggested some determinants for each dimension. After adjustment for age, however, nearly all associations were attenuated and rendered statistically non-significant. Thus, the age of MAs largely explained our initial observations. Only the following few associations persisted: having a high salary (≥2000€) versus the lowest salary category (≤1499€) related to a greater need for task-related independence (OR = 1.59, 95%-CI: 1.16–2.20) as well as a greater need for reward from the supervisor (OR = 1.53, 95%-CI: 1.12–2.09). After age adjustment also the association between having a leadership position and the need of greater task-related independence remained significant (OR = 1.78, 95%-CI: 1.37–2.32). Similar associations were found for the number of MAs in a practice and higher desire for rewards from the supervisor (OR = 1.60, 95%-CI: 1.13–2.26). Both, the increasing number of practitioners in a practice (OR for ≥3 practitioners versus 1 practitioner = 0.60, 95%-CI: 0.44–0.94) as well as the overall practice size (OR for 6–10 employees versus 1–5 employees = 0.58, 95%-CI: 0.40–0.98 and ≥ 11 employees compared to 1–5 employees = 0.60, 95%-CI: 0.41–0.91) were associated with decreasing reported needs regarding the working conditions.

Finally, the ordinal regression models with backward selection were run. Associations were only found for one single outcome variable, that is, task-related independence (the other data are not shown). Specifically, we found that the longer a MA worked in the MA profession the more task-related independence appeared to be needed (OR for 11–20 years versus ≤10 years = 2.12, 95%-CI: 1.37–3.28; OR for ≥21 years versus ≤10 years: OR = 2.20, 95%-CI: 1.46–3.32). Further, the odds of expressing a need for greater task-related independence were higher when one reported to hold a leadership position versus not (OR = 1.64, 95%-CI: 1.16–2.33).

## Discussion

To our knowledge, this is the first study to explicitly examine MAs’ desired improvements regarding their working conditions. We found that MAs generally reported high levels of such needs. Specifically, the need for higher reward from the supervisor, in terms of a higher salary, more recognition from society and more recognition and understanding from the supervisor, was mentioned frequently. Additionally, educational opportunities related to organizational leadership for supervising physicians represented a highly prevalent need. Needs pertaining to less documentation and less multitasking at work were also often stated. Further, our study identified three dimensions of needs, which captured working conditions, rewards, task-related independence. While we examined a large set of potential determinants of those different dimensions of needs, we found only very few associations.

### Findings in light of prior studies

Our study results are in line with previous studies, showing that across all inquired aspects, the satisfaction with income as well as the perceived recognition for their work from society is rather low among MAs [24, 25, 28]. All those earlier studies used a seven point Likert-scale (from 1 = extreme dissatisfaction to 7 = extreme satisfaction) to collect data on work satisfaction. Summarizing all three studies [24, 25, 28], MAs reported their satisfaction with income with mean values of 4.62 (SD = 1.68) [28], 3.44 (SD = 1.9) [25] and 3.89 (SD = 1.79) [24]. Since the nature of data from those studies is different from data on expressed needs, as gathered in our study, a comparison of those results cannot be performed easily. Nevertheless, it seems plausible that a low rating of one’s satisfaction with income translates into the experience of a need for higher income. While some of the needs expressed in our study may be rather specific for the MA occupation (e.g., to independently advise patients about their disease, make home visits etc.), there may be needs, which are likely prevalent across many types of occupations (e.g., the need for a higher salary). The issue of a low salary remains a crucial aspect nonetheless: The average monthly gross salary of fulltime employees in the service sector in Germany has amounted to 3,719€ in 2017 [29]. MAs usually receive a gross salary far below that average. In our study, for instance, only 58.2% of MAs working full-time reported to receive a gross salary of at least 2,000€. Close associations of low income with poor health have repeatedly been documented [30–32] and should be considered when addressing needs to improve the working life of MAs.

Desired work-related improvements concerning organizational processes such as better organization of practice/clinical procedures as well as the availability of more staff at the workplace were reported frequently. The challenges of staff shortage are widely reported throughout medical professions (e.g. nurses) due to economical restrictions and shortage of qualified personal [33].

Furthermore, previous studies addressed one’s opportunities (or lack thereof) to choose one’s own strategies to fulfill occupational tasks [24, 25, 28, 34] and the influence at work (e.g. the amount and content of work) of MAs [34]. All studies showed that MAs are dissatisfied with their freedom and opportunities to choose their preferred working strategies, and this dissatisfaction was specified in greater detail in our study: we found that MAs wanted less multitasking, which indicates that MAs cannot adopt their own working routines and have to fulfill several tasks at the same time. Additionally, most of the MAs in our study reported the need for less documentation. Earlier studies used instruments, which are not MA-specific, i.e. the Warr–Cook–Wall job satisfaction scale [24, 25, 28] as well as the survey of organizational attributes for primary care [24] and the Copenhagen Psychosocial Questionnaire (COPSOQ) [34]. In one study, those instruments were supplemented by a few questions regarding training facilities [25]. In contrast, we developed a questionnaire based on in-depth discussions with MAs, which specifically inquired after the improvements they felt to need. Likely, this has contributed to the completeness of our tool (i.e. all relevant aspects were measured). Accordingly, we contribute novel items measuring relevant aspects that have not yet been considered. Among those aspects are the need related to the reduction of documentation duties in day-to-day work, which ranked second in terms of frequency (76.0%). Another aspect that has been reported by more than half of the MAs (55.7%) pertained to the need for more staff at the workplace. Some studies already included the quality of leadership of the supervising physicians of MAs [24, 34]. Our study complements that evidence by finding that three-quarter of the respondents wished for special educational opportunities for their supervising physician related to organizational leadership skills.

Needs related to the reduction of specific demands (e.g., multitasking) combined with needs for increased rewards from the supervisor (e.g., income and recognition) are in keeping with the theoretical framework underlying the effort-reward-imbalance (ERI) model. The ERI model, which is (among other models, e.g., job-demand-control model [35], model of organizational justice [36]) a well-established theoretical model in work stress research, assumes that work stress is likely experienced when one’s perceived effort exceeds the rewards an employee experiences at work [37]. The ERI questionnaire has frequently been used to measure psychosocial working conditions in different occupational groups. In a previous report from our study, we found that almost two thirds of the MAs reported work stress according to the ERI model [9]. As previous research has repeatedly shown, high ERI levels are associated with poorer self-reported health, depressive symptoms and anxiety levels in health care professionals, reduced quality of patient care as well as the intention to leave one’s profession [9, 38–40]. Among MAs, in particular reward in terms of recognition from the supervisor seems to have an important role in this context, as our study suggests. Lacking support and recognition from the supervisor is strongly associated with an increased risk of burnout and decreasing job satisfaction [41]. Since mental illnesses are highly prevalent in health care professionals [42], social support from the supervisor may reduce the risk of poor mental health [43]. Furthermore, poor quality of leadership is negatively associated with job satisfaction [34] as well as the intention to leave one’s profession [4, 24, 44]. In our study, most of the MAs reported the need for educational opportunities for supervisors to develop/improve their leadership skills. Addressing the expressed needs may not only be important to attract or retain an adequately sized and skilled MA workforce, but is also relevant to the MAs’ health as well as to patients’ safety [9].

The final logistic regression model with backward selection showed only few significant associations, and those were limited to the outcome variable “task-related independence”. Specifically, the longer MAs had been working in the MA occupation, the higher was the reported need for more task-related independence. One approach to increasing work-related independence is to engage into ongoing education [45]. About 35% of our study participants had already taken part in further training courses, and 55% wanted more training opportunities. In another previous study among MAs [25], 90% of a total of 410 medical assistants working at a general practitioner’s practice had taken part in further training in the past three years, mainly related to quality management or vaccination assistance. Further, in that study MAs explained to take part in further training because of general interest (85.2%), to develop professionally (74.5%) as well as to keep up with developments in the practice (53.6%). The high motivation for further training should be supported by supervisors financially, but also through subsequent delegation of appropriate tasks to MAs. Due to their higher qualification and performance of additional or more complex tasks, supervisors are also to increase the salary of MAs accordingly. One may speculate that this consequence may be a reason for physicians not to support further training of MAs [7].

In summary, the three identified work-related dimensions are partly comparable to the three dimensions of the self-determination theory (SDT) (i.e., competence, relatedness and autonomy). Whereas we found the dimensions “working conditions”, “reward from the supervisor” and “task-related independence”, the SDT conceptualizes the fulfillment of basic psychological needs for “competence”, “relatedness” and “autonomy”, respectively, for being optimally motivated and experience well-being at the workplace [46]. While the SDT attributes great meaning to the “autonomy”, the items making up the the dimension “task-related independence”, have not been highly prevalent in the present study. One explanation may be that the needs regarding task-related independence are partly double-edged, that is, on the one hand there is hope for receiving more responsibility with potentially higher remuneration versus on the other hand the concern of increasing stress levels (e.g. expanded working hours when doing home visits) with possibly no increased remuneration.

As a previous study found, the fulfillment of the needs included in the SDT is associated with the personal-environmental fit (P-E fit) theory [47]. The P-E fit theory describes the (mis)match between characteristics of individuals and their work environments [16]. One can therefore assume, that reporting (numerous) needs may indicate a P-E misfit, which may lead to burnout and turnover intentions [48].

### Implications

Based on our findings, various starting points for interventions to improve the working experience of MAs can be identified. Previous studies among nurses which are in a profession somewhat comparable to MAs in terms of patient contact and many other working conditions found, for instance, that nurses mainly consider individual-level strategies instead of organizational-level strategies when facing work stress, that is, reliance on adjustment of their emotional, cognitive or behavioral self-regulation and functioning to improve their occupational experiences. Another strategy among nurses is to either change the employer or to even leave the profession [6]. Exit from the profession would of course not improve the working conditions of the remaining nurses (or in our study MAs) because of the reported lack of staff at their workplaces. We found in a prior qualitative study that MAs do not feel that there are any preventive strategies or coping strategies that can be applied during working hours besides to simply continue with their occupational tasks one after the other [7]. Studies among nurses might suggest preventive approaches that are potentially useful for MAs, since availability of data for MAs is currently low. A study among nurses found that they apply so-called selection, optimization and compensation (SOC) strategies to cope with their work conditions [49, 50]. These strategies aim for an efficient use of (remaining) individual resources. In order to ensure the maintenance of functioning and the minimization of losses, the selection of functional domains on which to focus one’s resources, the optimization of existing potential and the compensation for losses are at the core of these strategies [49, 50]. Evidence showed that using SOC strategies at work is conducive to nurses’ job performance under high workload levels and can help employees compensate for resource losses, resulting in higher work ability [51]. Nevertheless, it is suggested that these strategies are only effective within a supportive work environment and on an organizational level [50, 51]. Therefore, in the light of our study, MAs are unable to fully attain their reported desired work-related improvements on their own, but are dependent on external support. Another set of individual strategies relates to “job crafting” [52] (i.e. the redesign of work characteristics initiated by the employees themselves), which can be potentially useful to adjust job demands or to increase job resources. This implies that health care professionals might take the initiative to ask for support, feedback and training [53]. MAs therefore might be able to shape their working conditions to a certain extent with redesigning work characteristics and enhancing job resources.

Other strategies, which could be effective in improving working conditions and staff retention, could address organizational aspects. In this study most of the reported needs were structural or organizational (e.g., income, documentation effort, multitasking, staff) and thus only partly amendable to intervention by the MAs themselves (e.g. through SOC-strategies). Especially, the income of MAs has already been discussed as a precarious employment characteristic. Salary increases in collective wage agreements have been initiated with the hope of increasing the attractiveness of the profession [54]. Still, only few registered doctors comply with those agreements and simply pay the minimum wage [6]. To improve this situation of MAs, it needs to be ensured that all medical practices and clinics comply with the collective wage agreements. Alternatively, the minimum wage (currently at € 9.19 per hour in Germany) should be raised through legislative action. Another aspect would be to allow specialized MAs to bill the specific health care services they provide. In Germany, MAs can take part in training courses as so called care assistants in physicians’ practices (VERAH®) which is supposed to reduce the workload of the general practitioners [25]. In comparison to MAs, VERAHs additionally monitor and coordinate services, prevention management, case management and home visits on their own. Increasing the opportunities to account for services done by non-physician health care professionals could lead to an additional income for MAs [25]. One needs to bear in mind though that while such expansion of the responsibilities of MAs may increase their income, it will likewise increase their overall workload.

In addition to income, some other improvement needs could be met or reduced through MAs’ supervisors. In our study, most of the MAs reported to need more understanding and appreciation from their supervisor as well as educational opportunities related to organizational leadership for physicians. The Federal Medical Association (“Bundesärztekammer”) in Germany has developed a curriculum *medical leadership* which is designed for medical professionals from inpatient and outpatient care in leadership positions, but which is not mandatory for future physicians or practitioners. So far, different kinds of leadership development programs for physicians with the aim of an increased self-assessed knowledge and expertise have been evaluated [55]. It may be recommendable not to limit such development programs to physicians, but to improve the understanding and communication across professional groups through the inclusion of MAs [55]. In addition to physician-specific leadership development programs, so-called health circles may be implemented. Within these discussion groups, physicians and MAs can take shared responsibility in identifying problems and strategies to solve those problems on an organizational level [56]. Further, highly context-specific interventions strategies may be developed jointly by MAs and their supervisors with the help of external facilitators. Previous studies found that leadership is directly and indirectly affecting employees’ health at the workplace. Leaders may influence the working climate, promote healthy work designs or function as a role-model regarding healthy behavior [57]. Furthermore, poor leadership seems to be associated with adverse health outcomes among the subordinates (e.g. burnout) [58–61]. The special working relationship of MAs with physicians, in particular in small outpatient care practices, presents a challenge though: since there are no employee representatives in outpatient practices with few MAs (in contrast to most inpatient clinics), MAs would have to contact or criticize their supervisor directly, who is also their employer and of whom they are thus financially dependent. This might be a reason why MAs rarely join a professional association or insist on changing working conditions [6].

Overall, our study did not identify compelling determinants of needs among MAs, which implies that there are no specific subgroups that have to be taken into account when planning interventions. Instead, all MAs should be targeted. Work stress may be interpreted as one reason for the development of work-related improvement needs. A previous study of Viehmann et al. [8] examined the associations between stress and practice characteristics as well as individual characteristics and showed similar results. Only age and work experience, as interrelated factors, were associated with stress. Therefore, the study also indicates a need for interventions addressing the entire MA population and not specific subgroups. To identify which specific work-related intervention needs might be prevalent within an individual practice, the implementation of risk assessments, which are mandatory for employers in Germany and other western countries [62], is desirable.

### Strengths and limitations

Since our data is based on a cross-sectional study design, statements on causal relationships cannot be made. However, causality is not important for the interpretation of the prevalence of desired work-related improvements. Since the study population was recruited through various pathways, the number of contacted MAs is not known and therefore no response rate can be estimated. Additionally, selection bias cannot be excluded, since study participants might have been more (dis)satisfied or interested than other MAs, who did not participate. Our instrument captured needs, which have been expressed in prior qualitative interviews. This feature maximized the likelihood that we measured the full scope of needs that matter to MAs. While some MAs might have reported any needs they have, others may only have reported needs, which they thought would be susceptible to intervention. This could either lead to over- or underestimation of needs. Additionally, while the questionnaire and its response options have been developed to capture needs of MAs with high validity, the response format is not ideal for exploratory factor analysis, as only two response categories were and a higher number had been preferable for psychometric analyses. Consequently, our EFA was based on a tetrachoric matrix which is nevertheless a suitable approach [22]. Finally, as the EFA suggested factors with only three to five items, the internal consistency was somewhat low (Cronbach’s α between 0.61 and 0.70), but nevertheless seems acceptable given the low number of items and restricted score range [63]. Regarding the representativeness of our study population, the population characteristics are comparable to those of a representative study among MAs in Germany [8]. Further, the characteristics of the recruited study population were almost identical to the corresponding characteristics according to data from Federal Statistical Office on the MA population in Germany (i.e., age, gender and working arrangements) [64].

## Conclusions

Our study showed high prevalence rates of desired work-related improvements among medical assistants. In particular, needs regarding reward from the supervisor, such as income and recognition, were frequently mentioned and should be considered when planning interventions. Since the needs are distributed irrespectively of sociodemographic or practice-related characteristics, interventions should target and reach all MAs.

## Additional files

Additional file 1: Associations of sociodemographic and practice-relevant determinants with working conditions (ordinal logistic regression) (DOC 62 kb)

Additional file 2: Associations of sociodemographic and practice-relevant determinants with reward from the supervisor (ordinal logistic regression) (DOC 63 kb)

Additional file 3: Associations of sociodemographic and practice-relevant determinants with task-related independence (ordinal logistic regression) (DOC 63 kb)

## Data Availability

The datasets used and/or analyzed during the current study are available from the corresponding author on reasonable request.

## Abbreviations

CI: confidence interval
COPSOQ: Copenhagen Psychosocial Questionnaire
e.g.: example given
EFA: exploratory factor analysis
ERI: effort-reward imbalance
i.e.: in other words
MA: medical assistant
OR: odds ratio
P-E fit: person-environment fit
SD: standard deviation
SDT: self-determination theory
SOC: selection, optimization and compensation
VERAH: care assistants in physicians’ practices

## References

[1] Anderson A, Proudfoot JG, Harris M (2009). Medical assistants. A primary care workforce solution?. Aust Fam Physician.

[2] Laux G, Szecsenyi J, Mergenthal K, Beyer M, Gerlach F, Stock C (2015). Hausarztzentrierte Versorgung in Baden-Württemberg. Ergebnisse einer qualitativen und querschnittlich quantitativen Evaluation. Bundesgesundheitsbl..

[3] Gensichen J, Guethlin C, Sarmand N, Sivakumaran D, Jäger C, Mergenthal K (2012). Patients’ perspectives on depression case management in general practice – a qualitative study. Patient Educ Couns.

[4] Szecsenyi J, Goetz K, Campbell S, Broge B, Reuschenbach B, Wensing M (2011). Is the job satisfaction of primary care team members associated with patient satisfaction?. BMJ Qual Saf.

[5] Hall L, Johnson J, Watt I, Tsipa A, O'Connor D (2016). Healthcare staff wellbeing, burnout, and patient safety: a systematic review. PLoS One.

[6] Kathmann T, Dingeldey I (2013). Prekarisierung berufsfachlich qualifizierter Beschäftigung? Eine Analyse der Arbeitsbedingungen von medizinischen Fachangestellten.

[7] Vu-Eickmann P, Loerbroks A (2017). Psychosoziale Arbeitsbedingungen Medizinischer Fachangestellter: Ergebnisse einer qualitativen Studie zu den berufsspezifischen Belastungen, Ressourcen, Präventionsmöglichkeiten und Interventionsbedürfnissen. [psychosocial working conditions of physician assistants: results from a qualitative study on occupational stress, resources, possible approaches to prevention and intervention needs]. Z Evid Fortbild Qual Gesundhwes.

[8] Viehmann A, Kersting C, Thielmann A, Weltermann B (2017). Prevalence of chronic stress in general practitioners and practice assistants: personal, practice and regional characteristics. PLoS One.

[9] Vu-Eickmann P, Li J, Müller A, Angerer P, Loerbroks A (2018). Associations of psychosocial working conditions with health outcomes, quality of care and intentions to leave the profession: results from a cross-sectional study among physician assistants in Germany. Int Arch Occup Environ Health.

[10] Angerer P, Petru R, Nowak D, Weigl M (2008). Working conditions and depression in physicians. Dtsch Med Wochenschr.

[11] Wallace JE, Lemaire JB, Ghali WA (2009). Physician wellness: a missing quality indicator. Lancet..

[12] Karweina D (2013). Aggressiva Patienten konsequent "erziehen". Der Allgemeinarzt.

[13] Croidieu S, Charbotel B, Vohito M, Renaud L, Jaussaud J, Bourboul C (2008). Call-handlers' working conditions and their subjective experience of work: a transversal study. Int Arch Occup Environ Health.

[14] Furnham A, Schaeffer R (1984). Person-environment fit, job satisfaction and mental health. J Occup Organ Psychol.

[15] Green LW, Kreuter MW (2005). Health program planning: an educational and ecological approach. 4. Revised edition ed: McGraw-hill education ltd.

[16] Edwards JR, Caplan RD, Harrison RV, Cooper CL (1998). Person-environment fit theory: conceptual foundations, empirical evidence, and directions for future research. Theories of organizational stress.

[17] Asadi-Lari M, Packham C, Gray D (2003). Need for redefining needs. Health and quality of life outcomes.

[18] Wright J, Rhys W, Wilkinson JR (1998). Development and importance of health needs assessment. BMJ..

[19] Bradshaw J, McLachlan G (1972). Taxonomy of social need. Problems and progress in medical care : essays on current research. 7.

[20] Loerbroks A, Leucht V, Keuneke S, Apfelbacher CJ, Sheikh A, Angerer P (2016). Patients' needs in asthma treatment: development and initial validation of the NEAT questionnaire. J Asthma.

[21] Lozano LM, García-Cueto E, Muñiz J (2008). Effect of the number of response categories on the reliability and validity of rating scales. Methodology..

[22] Gadermann AM, Guhn M, Zumbo BD. Estimating ordinal reliability for Likert-type and ordinal item response data: a conceptual, empirical, and practical guide. Practical Assessment. Research a& Evaluation. 2012;17(3).

[23] Wirth RJ, Edwards MC (2007). Item factor analysis: current approaches and future directions. Psychol Methods.

[24] Gavartina A, Zaroti S, Szecsenyi J, Miksch A, Ose D, Campbell SM, et al. Practice assistants in primary care in Germany – associations with organizational attributes on job satisfaction. BMC Fam Pract. 2013;(110):14. 10.1186/1471-2296-14-110 PMC3750430 23915225

[25] Mergenthal K, Banduhn S, Gerlach I, Marini A, März B (2014). Müller V, et al. [dedicated but poorly paid! Study on Health care assistants’ views on their profession]. Z Allg Med.

[26] Dunkler D, Plischke M, Leffondré K, Heinze G (2014). Augmented backward elimination: a pragmatic and purposeful way to develop statistical models. PLoS One.

[27] Cohen J (1988). Statistical power analysis for the behavioral sciences.

[28] Goetz K, Campbell S, Broge B, Brodowski M, Steinhaeuser J, Wensing M (2013). Job satisfaction of practice assistants in general practice in Germany: an observational study. Fam Pract.

[29] Statistisches Bundesamt (Destatis). Verdienste und Arbeitskosten. Arbeitnehmerverdienste. 2018.

[30] Wood AM, Boyce CJ, Moore SC, Brown GDA (2012). An evolutionary based social rank explanation of why low income predicts mental distress: a 17 year cohort study of 30,000 people. J Affect Disord.

[31] Martikainen P, Adda J, Ferrie JE, Davey Smith G, Marmot M (2003). Effects of income and wealth on GHQ depression and poor self rated health in white collar women and men in the Whitehall II study. J Epidemiol Community Health.

[32] Lynch JW, Kaplan GA (1997). Understanding how inequality in the distribution of income affects Health. J Health Psychol.

[33] Reifferscheid A, Pomorin N, Wasem J (2016). Nursing care deficits in German hospitals – results of a Nationwide survey of supervisory staff in hospital. Gesundheitsw..

[34] Goetz K, Berger S, Gavartina A, Zaroti S, Szecsenyi J. How psychosocial factors affect well-being of practice assistants at work in general medical care? – a questionnaire survey. BMC Fam Pract. 2015;16(166). 10.1186/s12875-015-0366-y PMC4642745 26560752

[35] Karasek RA (1979). Job demands, job decision latitude, and mental strain: implications for job redesign. Administrative Science Quaterly.

[36] Colquitt JA, Conlon DE, Wesson MJ, Porter CO, Ng KY (2001). Justice at the millennium: a meta-analytic review of 25 years of organizational justice research. J Appl Psychol.

[37] Siegrist J (1996). Adverse health effects of high-effort/low-reward conditions. J Occup Health Psychol.

[38] Angerer P, Weigl M. Physicians' psychosocial work conditions and quality of care: a literature review. Professions and Professionalism. 2015;5(1).

[39] Klein J, Grosse Frie K, Blum K, Siegrist J, von dem Knesebeck O (2010). Effort-reward imbalance. Job Strain and Burnout among Clinicians in Surgery Psychother Psych Med.

[40] Li J, Galatsch M, Siegrist J, Müller BH, Hasselhorn HM (2011). Reward frustration at work and intention to leave the nursing profession—prospective results from the European longitudinal NEXT study. Int J Nurs Stud.

[41] Hämmig O (2017). Health and well-being at work: the key role of supervisor support. SSM - Population Health.

[42] Limbrecht-Ecklundt Kerstin, Flaig Tanja, Gündel Harald, Traue Harald C, Jerg-Bretzke Lucia (2014). Psychische Gesundheit und Arbeitsbelastung bei Ärztinnen und Ärzten. Prävention und Gesundheitsförderung.

[43] Woodhead EL, Northrop L, Edelstein B (2016). Stress, social support, and burnout among long-term care nursing staff. J Appl Gerontol.

[44] Cowden T, Cummings G, Profetto-Mcgrath J (2011). Leadership practices and staff nurses’ intent to stay: a systematic review. J Nurs Manag.

[45] Redaèlli M, Bassüner S, Teschner D, Stock S (2014). Practice nurses can do more - online surveys of VERAH-graduates and practice owners. Z Allg Med.

[46] Van den Broeck A, Ferris DL, Chang C-H, Rosen CC (2016). A review of self-determination Theory’s basic psychological needs at work. J Manag.

[47] Greguras GJ, Diefendorff JM (2009). Different fits satisfy different needs: linking person-environment fit to employee commitment and performance using self- determination theory. J Appl Psychol.

[48] Andela M, van der Doef M. A comprehensive assessment of the person–environment fit dimensions and their relationships with work-related outcomes. J Career Dev. 2018.

[49] von Bonsdorff ME, von Bonsdorff MB, Zhou ZE, Kauppinen M, Miettinen M, Rantanen T (2014). Organizational justice, selection, optimization with compensation, and nurses' work ability. J Occup Environ Med.

[50] Baethge A, Müller A, Rigotti T (2016). Nursing performance under high workload: a diary study on the moderating role of selection, optimization and compensation strategies. J Adv Nurs.

[51] Müller A, Weigl M, Heiden B, Glaser J, Angerer P. Promoting work ability and well-being in hospital nursing: the interplay of age, job control, and successful ageing strategies. Work. 2012:5137–44. 10.3233/WOR-2012-0083-5137 22317516

[52] Wrzesniewski A, Dutton JE (2001). Crafting a job: Revisioning employees as active crafters of their work. Acad Manag Rev.

[53] Bakker AB (2017). Job crafting among health care professionals: the role of work engagement. Nurs Manag.

[54] Bistrup R (2011). Mehr Geld für Berufsanfänge. Dtsch Arztbl.

[55] Frich JC, Brewster AL, Cherlin EJ, Bradley EH (2014). Leadership development programs for physicians: a systematic review. J Gen Intern Med.

[56] Bourbonnais R, Brisson C, Vinet A, Vézina M, Abdous B, Gaudet M (2006). Effectiveness of a participative intervention on psychosocial work factors to prevent mental health problems in a hospital setting. Occup Environ Med.

[57] Wegge J, Shemla M, Haslam A (2014). Leader behavior as a determinant of health at work: specification and evidence of five key pathways. Ger J Hum Resour Manag.

[58] Laschinger HKS, Fida R (2014). New nurses burnout and workplace wellbeing: the influence of authentic leadership and psychological capital. Burnout Research.

[59] Schmidt B, Loerbroks A, Herr RM, Wilson MG, Jarczok MN, Litaker D (2014). Associations between supportive leadership and employees self-rated Health in an occupational sample. Int J Behav Med.

[60] Schmid JA, Jarczok MN, Sonntag D, Herr RM, Fischer JE, Schmidt B (2017). Associations between supportive leadership behavior and the costs of absenteeism and Presenteeism. An Epidemiological and Economic Approach JOEM.

[61] Schmidt B, Herr RM, Jarczok MN, Baumert J, Lukaschek K, Emeny RT (2018). Lack of supportive leadership behavior predicts suboptimal self-rated health independent of job strain after 10 years of follow-up: findings from the population-based MONICA/KORA study. Int Arch Occup Environ Health.

[62] Sakowski P, Marcinkiewicz A. Health Promotion and Prevention in Occupational Health Systems in Europe. International Journal of Occupational Medicine and Environmental Health 209;32(3). 10.13075/ijomeh.1896.01384 30356227

[63] Cortina JM (1993). What is coefficient alpha? An examination of theory and applications. J Appl Psychol.

[64] Statistisches Bundesamt. Anzahl der Beschäftigten im Gesundheitswesen 2015. Wiesbaden; 2017.

