# Supplementary material for: Desired improvements of working conditions among medical assistants in Germany: a cross-sectional study
Source: J Occup Med Toxicol. 2019 Jun 1;14:18. doi: 10.1186/s12995-019-0237-x (PMC6545209; doi:10.1186/s12995-019-0237-x)
Supplement: Supplementary file 1 — Associations of sociodemographic and practice-relevant determinants with working conditions (ordinal logistic regression) (DOC 62 kb) [file 12995_2019_237_MOESM1_ESM.doc]

Additional file 1: Associations of sociodemographic and practice-relevant determinants with working conditions (ordinal logistic regression)

| Variable | | Unadjusted | | Age-adjusted | |
| --- | --- | --- | --- | --- | --- |
|  |  | OR | CI | OR | CI |
| Age (years) | 18-35 | 1.00 | Ref. | - | - |
|  | 36-45 | 1.17 | 0.80-1.71 | - | - |
|  | ≥ 46 | 1.01 | 0.68-1.49 | - | - |
| Marital status | single | 1.00 | Ref. | 1.00 | Ref. |
|  | Married/partnership | 1.05 | 0.77-1.42 | 1.04 | 0.74-1.46 |
| Gross salary (€) | ≤ 1499 | 1.00 | Ref. | 1.00 | Ref. |
|  | 1500-1999 | 0.96 | 0.64-1.44 | 0.97 | 0.64-1.47 |
|  | ≥ 2000 | 0.94 | 0.66-1.34 | 0.97 | 0.67-1.39 |
| Years in Job | ≤ 10 | 1.00 | Ref. | 1.00 | Ref. |
|  | 11-20 | 1.06 | 0.71-1.56 | 0.88 | 0.49-1.57 |
|  | ≥ 21 | 0.93 | 0.64-1.36 | 0.74 | 0.38-1.44 |
| Practice type | Specialist | 1.00 | Ref. | 1.00 | Ref. |
|  | General practitioner | 1.11 | 0.79-1.56 | 1.21 | 0.80-1.58 |
| Employment status | Part-time/Mini-job | 1.00 | Ref. | 1.00 | Ref. |
|  | Full-time | 1.19 | 0.86-1.63 | 1.25 | 0.89-1.76 |
| Leadership position | No | 1.00 | Ref. | 1.00 | Ref. |
|  | Yes | 1.06 | 0.78-1.45 | 1.10 | 0.80-1.50 |
| Number of MAs (n) | 1-3 | 1.00 | Ref. | 1.00 | Ref. |
|  | 4-6 | 0.72 | 0.50-1.04 | 0.74 | 0.51-1.07 |
|  | ≥ 7 | 0.68 | 0.50-1.02 | 0.70 | 0.46-1.04 |
| Number of practitioners (n) | 1 | 1.00 | Ref. | 1.00 | Ref. |
|  | 2 | 0.79 | 0.53-1.17 | 0.83 | 0.56-1.23 |
|  | ≥ 3 | 0.60 | 0.41-0.88 | 0.64 | 0.44-0.94 |
| Practice size (n) | 1-5 | 1.00 | Ref. | 1.00 | Ref. |
|  | 6-10 | 0.58 | 0.39-0.89 | 0.63 | 0.40-0.98 |
|  | ≥ 11 | 0.60 | 0.39-0.93 | 0.61 | 0.41-0.91 |
| Practice location | Countryside | 1.00 | Ref. | 1.00 | Ref. |
|  | Small city | 0.88 | 0.58-1.34 | 0.85 | 0.56-1.30 |
|  | Major city | 0.91 | 0.59-1.39 | 0.89 | 0.58-1.36 |
