# Supplementary material for: Desired improvements of working conditions among medical assistants in Germany: a cross-sectional study
Source: J Occup Med Toxicol. 2019 Jun 1;14:18. doi: 10.1186/s12995-019-0237-x (PMC6545209; doi:10.1186/s12995-019-0237-x)
Supplement: Supplementary file 2 — Associations of sociodemographic and practice-relevant determinants with reward from the supervisor (ordinal logistic regression) (DOC 63 kb) [file 12995_2019_237_MOESM2_ESM.doc]

Additional file 2: Associations of sociodemographic and practice-relevant determinants with Reward from the supervisor (ordinal logistic regression)

| Variable | | Unadjusted | | Age-adjusted | |
| --- | --- | --- | --- | --- | --- |
|  |  | OR | CI | OR | CI |
| Age (years) | 18-35 | 1.00 | Ref. | - | - |
|  | 36-45 | 1.30 | 0.94-1.79 | - | - |
|  | ≥ 46 | 1.43 | 1.03-1.97 | - | - |
| Marital status | single | 1.00 | Ref. | 1.00 | Ref. |
|  | Married/partnership | 1.02 | 0.79-1.32 | 0.87 | 0.65-1.17 |
| Gross salary (€) | ≤ 1499 | 1.00 | Ref. | 1.00 | Ref. |
|  | 1500-1999 | 1.00 | 0.71-1.42 | 1.40 | 0.73-1.47 |
|  | ≥ 2000 | 1.55 | 1.15-2.11 | 1.53 | 1.12-2.09 |
| Years in Job | ≤ 10 | 1.00 | Ref. | 1.00 | Ref. |
|  | 11-20 | 1.17 | 0.83-1.63 | 1.04 | 0.63-1.74 |
|  | ≥ 21 | 1.35 | 0.99-1.85 | 1.31 | 0.74-2.34 |
| Practice type | Specialist | 1.00 | Ref. | 1.00 | Ref. |
|  | General practitioner | 1.00 | 0.75-1.33 | 0.96 | 0.74-1.31 |
| Employment status | Part-time/Mini-job | 1.00 | Ref. | 1.00 | Ref. |
|  | Full-time | 1.07 | 0.82-1.40 | 1.23 | 0.93-1.64 |
| Leadership position | No | 1.00 | Ref. | 1.00 | Ref. |
|  | Yes | 1.23 | 0.95-1.60 | 1.19 | 0.91-1.55 |
| Number of MAs (n) | 1-3 | 1.00 | Ref. | 1.00 | Ref. |
|  | 4-6 | 1.13 | 0.83-1.55 | 1.18 | 0.86-1.62 |
|  | ≥ 7 | 1.53 | 1.09-2.16 | 1.60 | 1.13-2.26 |
| Number of practitioners (n) | 1 | 1.00 | Ref. | 1.00 | Ref. |
|  | 2 | 0.81 | 0.58-1.14 | 0.81 | 0.58-1.15 |
|  | ≥ 3 | 1.14 | 0.83-1.58 | 1.20 | 0.87-1.66 |
| Practice size (n) | 1-5 | 1.00 | Ref. | 1.00 | Ref. |
|  | 6-10 | 0.82 | 0.58-1.16 | 1.36 | 0.93-2.00 |
|  | ≥ 11 | 1.30 | 0.89-1.89 | 0.85 | 0.60-1.20 |
| Practice location | Countryside | 1.00 | Ref. | 1.00 | Ref. |
|  | Small city | 1.38 | 0.97-1.98 | 1.40 | 0.97-2.00 |
|  | Major city | 1.20 | 0.83-1.72 | 1.20 | 0.83-1.72 |
