# Supplementary material for: Desired improvements of working conditions among medical assistants in Germany: a cross-sectional study
Source: J Occup Med Toxicol. 2019 Jun 1;14:18. doi: 10.1186/s12995-019-0237-x (PMC6545209; doi:10.1186/s12995-019-0237-x)
Supplement: Supplementary file 3 — Associations of sociodemographic and practice-relevant determinants with task-related independence (ordinal logistic regression) (DOC 63 kb) [file 12995_2019_237_MOESM3_ESM.doc]

Additional file 3: Associations of sociodemographic and practice-relevant determinants with task-related independence (ordinal logistic regression)

| Variable | | Unadjusted | | Age-adjusted | |
| --- | --- | --- | --- | --- | --- |
|  |  | OR | CI | OR | CI |
| Age (years) | 18-35 | 1.00 | Ref. | - | - |
|  | 36-45 | 1.98 | 1.41-2.77 | - | - |
|  | ≥ 46 | 2.12 | 1.51-2.98 | - | - |
| Marital status | single | 1.00 | Ref. | 1.00 | Ref. |
|  | Married/partnership | 1.43 | 1.08-1.85 | 1.09 | 0.81-1.47 |
| Gross salary (€) | ≤ 1499 | 1.00 | Ref. | 1.00 | Ref. |
|  | 1500-1999 | 1.28 | 0.89-1.82 | 1.41 | 0.98-2.03 |
|  | ≥ 2000 | 1.65 | 1.21-2.25 | 1.59 | 1.16-2.20 |
| Years in Job | ≤ 10 | 1.00 | Ref. | 1.00 | Ref. |
|  | 11-20 | 1.89 | 1.33-2.67 | 1.69 | 1.00-2.88 |
|  | ≥ 21 | 2.20 | 1.58-3.06 | 2.38 | 1.30-4.35 |
| Practice type | Specialist | 1.00 | Ref. | 1.00 | Ref. |
|  | General practitioner | 1.05 | 0.78-1.40 | 1.05 | 0.78-1.41 |
| Employment status | Part-time/Mini-job | 1.00 | Ref. | 1.00 | Ref. |
|  | Full-time | 0.73 | 0.55-0.96 | 0.90 | 0.67-1.21 |
| Leadership position | No | 1.00 | Ref. | 1.00 | Ref. |
|  | Yes | 1.97 | 1.50-2.57 | 1.83 | 1.40-2.42 |
| Number of MAs (n) | 1-3 | 1.00 | Ref. | 1.00 | Ref. |
|  | 4-6 | 0.87 | 0.63-1.20 | 0.87 | 0.63-1.21 |
|  | ≥ 7 | 0.81 | 0.57-1.14 | 0.83 | 0.58-1.18 |
| Number of practitioners (n) | 1 | 1.00 | Ref. | 1.00 | Ref. |
|  | 2 | 0.91 | 0.64-1.29 | 0.95 | 0.67-1.35 |
|  | ≥ 3 | 0.91 | 0.66-1.27 | 1.00 | 0.72-1.40 |
| Practice size (n) | 1-5 | 1.00 | Ref. | 1.00 | Ref. |
|  | 6-10 | 0.82 | 0.58-1.16 | 0.88 | 0.59-1.31 |
|  | ≥ 11 | 0.83 | 0.57-1.22 | 0.84 | 0.57-1.16 |
| Practice location | Countryside | 1.00 | Ref. | 1.00 | Ref. |
|  | Small city | 1.10 | 0.76-1.59 | 1.09 | 0.75-1.60 |
|  | Major city | 1.36 | 0.94-1.98 | 1.34 | 0.92-1-97 |
